# Construction, alignment and analysis of twelve framework physical maps that represent the ten genome types of the genus *Oryza*

**DOI:** 10.1186/gb-2008-9-2-r45

**Published:** 2008-02-28

**Authors:** HyeRan Kim, Bonnie Hurwitz, Yeisoo Yu, Kristi Collura, Navdeep Gill, Phillip SanMiguel, James C Mullikin, Christopher Maher, William Nelson, Marina Wissotski, Michele Braidotti, David Kudrna, José Luis Goicoechea, Lincoln Stein, Doreen Ware, Scott A Jackson, Carol Soderlund, Rod A Wing

**Affiliations:** 1 Arizona Genomics Institute, Department of Plant Sciences, University of Arizona, Tucson, Arizona 85721, USA; 2 Cold Spring Harbor Laboratory, Cold Spring Harbor, New York 11724, USA; 3 Department of Agronomy, Purdue University, West Lafayette, Indiana 47907, USA; 4 Department of Horticulture, Purdue University, West Lafayette, Indiana 47907, USA; 5 Genome Technology Branch, NHGRI, National Institutes of Health, Bethesda, Maryland, 20892, USA; 6 Department of Biomedical Engineering, Stony Brook University, Stony Brook, New York 11794, USA; 7 Arizona Genomics Computational Laboratory, University of Arizona, Tucson, Arizona 85721, USA; 8 USDA-ARS NAA Plant, Soil and Nutrition Laboratory Research Unit, Ithaca, New York 14853, USA

## Abstract

We describe the establishment and analysis of a genus-wide comparative framework composed of 12 bacterial artificial chromosome fingerprint and end-sequenced physical maps representing the 10 genome types of *Oryza *aligned to the *O. sativa *ssp. *japonica *reference genome sequence. Over 932 Mb of end sequence was analyzed for repeats, simple sequence repeats, miRNA and single nucleotide variations, providing the most extensive analysis of *Oryza *sequence to date.

## Abstract 2

Bacterial artificial chromosome (BAC) fingerprint and end-sequenced physical maps representing the ten genome types of Oryza are presented

## Background

Comparative genomics is a powerful tool for unraveling the evolutionary history and gene functionality of related species. The availability of high resolution genetic and physical maps and genome assemblies has established comparative genomics platforms in organisms ranging from bacteria and fungi to animals and plants [1-7]. The majority of sequence-based eukaryotic platforms have focused on comparisons between genera [8-11]. Although highly informative, the divergence times between genera are often too distant to be useful for the identification of conserved noncoding sequences such as transcription factor binding sites, enhancers and matrix attachment regions (for example, see [12,13]). Sequence comparisons between species within a single genus have focused primarily on orthologous loci or genomic regions [14,15]. Whole genome comparative platforms within a genus are still in their infancy, with the most notable exception being the generation of whole genome shotgun assemblies of 12 *Drosophila *species that span an evolutionary time frame of approximately 60 million years [16].

In plants, rice (*Oryza sativa*) and thale cress (*Arabidopsis thaliana*) are important model systems for functional and evolutionary biology. Both genomes have been completely sequenced [17,18] and serve as the reference sequences for the major monocot and dicot plant lineages. Rice has added significance by virtue of being the world's most important crop, directly feeding half the human population. The population that depends on rice is expected to double in 25-50 years and there is thus an intense effort by breeders to double current rice yields using less land and water and on poorer soils [19]. To achieve this goal, the plant biology community is engaged in a concerted effort to functionally characterize all plant genes in both model plants using approaches including genetics, transgenetics, and comparative genomics. These efforts have significance not only to world food and bio-energy security issues but also to fundamental eukaryotic biology.

Comparative genomics across the cereals and within the genus *Oryza *will play a major role in these efforts. *Oryza *is composed of 24 species, including 2 domesticated (*O. sativa*, and *O. glaberrima*) and 22 wild species [20,21]. These species are classified into ten genome types (six diploids and four allotetraploids) based on crossing barriers [22,23], chromosome pairing [24,25], morphology [26,27], and molecular phylogenetics [28]. Compared to the domesticates, the wild relatives of rice are phenotypically inferior grass-like plants, but are a virtually untapped reservoir of agriculturally important genes and allelic variants that can be used to improve cultivated rice [21,29]. Therefore, comparative genomics of wild and domesticated *Oryza *species may not only provide useful materials for breeding, but will also shed light on the evolution and domestication of cultivated rice.

Our long-term objective is to develop a genome-level comparative experimental system for the genus *Oryza *(the *Oryza *Map Alignment Project (OMAP)). Our strategy is to develop bacterial artificial chromosome (BAC)-end sequence (BES) physical frameworks of the ten genome types of *Oryza *and align them to the reference sequence. These frameworks can then be populated with an array of phenotypic, genetic, biochemical, and physiological data to address fundamental questions in biology and agriculture. Previously, we described the construction and characterization of 12 BAC libraries from representative species that encompass the 10 genome types of *Oryza *[30]. Here we report the construction of 12 BAC/BES framework physical maps derived from these libraries and an analysis of the BESs in terms of transposable element, simple sequence repeat (SSR), microRNA (miRNA), and single nucleotide variation (SNV) content.

## Results

### Fingerprinting, BAC end sequencing and contig assembly to generate phase I physical maps for the ten genome types of *Oryza*

BAC-based physical maps for the ten genome types of *Oryza *were generated using a two-step procedure. First, clones from 12 *Oryza *BAC libraries [30] were fingerprinted [31] and BAC end sequenced. Fingerprints from each library were then assembled into contigs using FPC [32]. Fingerprint, BES and FPC assembly statistics are summarized in Tables 1 and 2. Briefly, we generated a total of 710,536 fingerprints and 1,452,912 BESs (approximately 932 Mb). Fingerprinting and BES success rates ranged from 85-94% and 88-96%, respectively. BESs represented approximately 8-17% of each *Oryza *genome, corresponding to one sequence tag per every 4-8 kb. From 90-98% of the BESs were paired and ranged in sequence length from 559 bp in *O. minuta *[BBCC] to 717 bp in *O. officinalis *[CC]. More importantly, an average of 89% of the fingerprinted clones had paired BESs. An average of 95% of the fingerprints assembled into contigs, with the remainder existing as singletons. The number of assembled contigs for each species ranged from 422 in *O. brachyantha *[FF] to 3,962 in *O. minuta *[BBCC], which roughly correlated with the size of each genome. The 12 unedited FPC assemblies are defined as phase I physical maps (Table 2) and are available interactively using WebFPC [33] or CMap, or as downloadable FPC files (see Materials and methods).

**Table 1:** Summary of BAC end sequences of 12 *Oryza *species

Species	Genome type	Genome size (Mb)*	No. of GenBank submissions	Average length after trim (in GenBank)	Total sequenced length (in GenBank)	Genome coverage	No. of forward reads	No. of reverse reads	No. of clones with paired reads (% of total BES)
*O. nivara*	AA	448	106,124	665 bp	~ 71 Mb	16%	53,450	52,674	51,820 (98%)
*O. rufipogon*	AA	439	70,982	704 bp	~ 50 Mb	11%	35,473	35,509	34,747 (98%)
*O. glaberrima*	AA	357	66,821	590 bp	~ 39 Mb	11%	33,456	33,365	30,885 (92%)
*O. punctata*	BB	425	68,384	710 bp	~ 49 Mb	11%	35,051	33,333	32,284 (94%)
*O. officinalis*	CC	651	101,091	717 bp	~ 72 Mb	11%	49,972	49,052	47,197 (93%)
*O. minuta*	BBCC	1,124	169,460	559 bp	~ 95 Mb	8%	85,466	83,994	82,248 (97%)
*O. alta*	CCDD	1,008	128,732	586 bp	~ 75 Mb	7%	64,365	64,367	58,217 (90%)
*O. australiensis*	EE	965	135,769	625 bp	~ 85 Mb	9%	67,245	68,524	64,081 (94%)
*O. brachyantha*	FF	362	67,364	672 bp	~ 45 Mb	13%	34,009	33,355	32,258 (96%)
*O. granulata*	GG	882	138,171	674 bp	~ 93 Mb	11%	69,962	68,209	66,434 (96%)
*O. ridleyi*	HHJJ	1,283	204,729	632 bp	~ 129 Mb	10%	102,640	102,089	98,160 (96%)
*O. coarctata*	HHKK	771^†^	195,285	661 bp	~ 129 Mb	17%	100,341	94,944	91,853 (94%)

Total/Average			1,452,912	650 bp	~ 932 Mb	11%	731,430	719,415	690,184 (95%)

*From [30]. ^†^By K Arumuganathan using flow cytometric methods as previously described [30].

**Table 2:** Summary of phase I FPC physical maps of 12 *Oryza *species

Species	Average insert size	No. of total attempted	No. of clones FPCed	Genome coverage by all FPCed clones	Success ratio	Total organellar contam + no. inserts containing clones*	No. of FPC clones with paired BES reads	No. of singletons (%)	No. of contigs	Total CB units	Average no. of bands/clone	Deduced size of 1 CB unit^† ^(kb)	Deduced genome size (Mb)^‡ ^(coverage)
*O. nivara*	161	55,296	51,056	18	92%	4.1%	48,032 (94%)	2,356 (4.6%)	456	384,733	121.6	1.32	509 (114%)
*O. rufipogon*	134	35,712	33,023	10	92%	3.9%	31,202 (94%)	1,305 (4.0%)	637	406,768	106.2	1.26	513 (117%)
*O. glaberrima*	130	36,864	33,065	12	90%	3.7%	27,661 (84%)	2,098 (6.3%)	905	309,740	103.9	1.25	388 (109%)
*O. punctata*	142	36,864	34,224	11	93%	1.8%	30,228 (88%)	1,482 (4.3%)	490	340,240	117.4	1.21	412 (97%)
*O. officinalis*	141	54,144	45,856	10	85%	6.7%	40,549 (88%)	1,133 (2.5%)	703	462,126	115.5	1.22	564 (87%)
*O. minuta*	125	92,160	86,861	10	94%	1.2%	77,999 (90%)	9,576 (11.0%)	3,962	1,004,697	96.6	1.29	1,300 (116%)
*O. alta*	133	73,728	63,860	8	87%	0.4%	50,842 (80%)	3,111 (4.9%)	2,492	870,411	109.3	1.22	1,059 (105%)
*O. australiensis*	152	73,728	67,416	11	91%	2.7%	58,992 (88%)	3,144 (4.7%)	1,003	691,155	125.1	1.22	840 (87%)
*O. brachyantha*	131	36,864	33,424	12	91%	1.7%	29,523 (88%)	2,354 (7.0%)	422	215,208	100.9	1.30	279 (77%)
*O. granulata*	134	73,728	64,836	10	88%	3.4%	58,818 (91%)	3,032 (4.7%)	2,358	1,078,237	120.8	1.11	1,196 (136%)
*O. ridleyi*	127	110,592	104,393	10	94%	2.1%	93,192 (89%)	5,169 (5.0%)	2,190	1,216,461	111.2	1.14	1,389 (108%)
*O. coarctata*	123	100,224	92,522	15	92%	2.1%	82,180 (89%)	1,810 (2.0%)	1,250	611,953	100.0	1.23	753 (98%)

Total/Average	136	779,904	710,536	11	91%	2.8%	629,218 (89%)	5.1%			110.7	1.23	

*From [30]. ^†^Deduced one CB unit size = average inset size/average number of bands. ^‡^Deduced genome size = ^†^Deduced one CB unit size × Total CB units.

To determine the approximate genome coverage of each of the 12 phase I physical maps, we estimated the size of the ordered consensus bands for each map in kilobases based on the average insert size of each BAC library and the average number of fingerprint bands observed for each species. Using this estimation, the genome coverage of the phase I physical maps ranged from 136% in *O. granulata *[GG] to 77% in *O. brachyantha *[FF]. In total, 7 of the physical maps had coverages greater that 100% (*O. nivara*, *O. rufipogon*, *O. glaberrima*, *O. minuta*, *O. alta*, *O. granulata*, and *O. ridleyi*), 4 had coverages between 98% and 87% (*O. punctata*, *O. officinalis*, *O. australiensis*, and *O. coarctata*) and 1 had a coverage of 77% (*O. brachyantha*). Coverage discrepancies above or below 100% could be due to a number of parameters, including: possible overlaps between the contigs that were not merged by the criteria used in this study; inaccurate estimation of genome size; and over-estimation of average insert sizes of the BAC libraries that would lead to an over-estimation of kilobases per consensus band (CB).

### Alignment of phase I physical maps to the *O. sativa *reference sequence

To evaluate the coverage of the phase I physical maps graphically, we aligned the BESs associated with each FPC contig to the *O. sativa *reference sequence (RefSeq) [18] using SyMAP [34]. SyMAP displays for all 12 physical maps can be viewed at [35] (display details are in Additional data file 1) and are summarized in Table 3 and Additional data file 2. Overall, between 61% and 95% of the contigs from each of the phase I physical maps could be aligned to the *O. sativa *RefSeq. Contigs not assigned a chromosomal position were generally small (133-289 CB/contig) and had few clones per contig (3-38 clones/contig) (Table 3). The percentage of BESs aligned, under identical parameters, was the least in *O. alta *[CCDD] (8% alignment) and the greatest in the AA genome type species *O. nivara *(60% alignment). Interestingly, the FF (*O. brachyantha*) and HHKK (*O. coarctata*) genome type species showed substantially more alignments to the *O. sativa *RefSeq than more closely related species (CC, BBCC, CCDD, EE, GG, and HHJJ).

**Table 3:** Summary of alignments of 12 OMAP Phase I FPC maps to the *O. sativa *RefSeq

								Not aligned contig	
								
Species	No. of contigs aligned	Total CB aligned	Total size aligned* (Mb)	No. of clones in aligned contig	No. of BESs aligned	Average identity of BES alignments	E-value of BES alignments	CB/contig (average)	No. of clones/contig (average)
*O. nivara*	368 (81%)	371,561 (97%)	490 (109%)	48,424 (99%)	63,293 (60%)	97%	e-237 to e-33	150	3
*O. rufipogon*	581 (91%)	399,346 (98%)	503 (115%)	31,398 (99%)	36,725 (52%)	97%	e-224 to e-57	133	6
*O. glaberrima*	860 (95%)	302,696 (98%)	378 (106%)	30,708 (99%)	33,356 (50%)	97%	e-213 to e-42	157	6
*O. punctata*	462 (94%)	332,224 (98%)	402 (95%)	31,674 (97%)	25,842 (38%)	93%	e-183 to e-30	286	38
*O. officinalis*	617 (88%)	448,477 (97%)	547 (84%)	44,194 (99%)	25,699 (25%)	93%	e-204 to e-16	159	6
*O. minuta*	3,073 (78%)	833,710 (83%)	1,075 (96%)	66,277 (86%)	46,227 (27%)	93%	e-173 to e-07	192	12
*O. alta*	1,571 (63%)	671,625 (77%)	819 (81%)	50,281 (83%)	10,770 (8%)	93%	e-193 to e-02	216	11
*O. australiensis*	787 (78%)	630,836 (91%)	770 (80%)	60,538 (94%)	20,429 (15%)	93%	e-182 to e-03	279	17
*O. brachyantha*	351 (83%)	205,608 (96%)	267 (74%)	30,717 (99%)	25,387 (38%)	91%	e-175 to e-18	135	5
*O. granulata*	1,444 (61%)	814,383 (76%)	904 (102%)	50,117 (81%)	18,785 (14%)	91%	e-179 to e-05	289	13
*O. ridleyi*	1,756 (80%)	1,123,902 (92%)	1,281 (100%)	94,565 (95%)	37,300 (18%)	92%	e-170 to e-06	213	11
*O. coarctata*	1,002 (80%)	579,809 (95%)	713 (92%)	89,775 (99%)	53,920 (28%)	92%	e-184 to e-07	130	4

*Deduced from [1 CB unit size from Table 2 × Total CB unit aligned].

**Figure 1:**
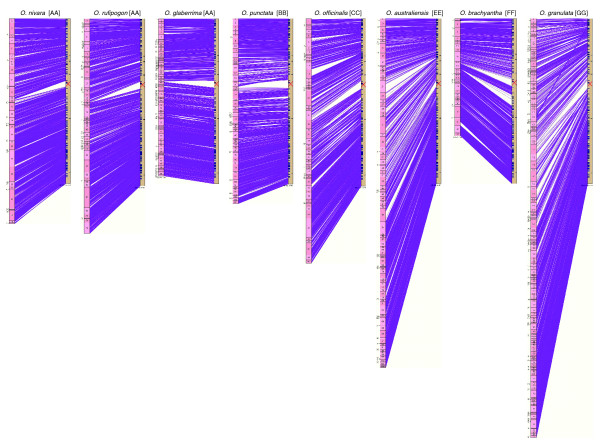
SyMAP view of unedited physical maps of chromosome 1 from eight diploid *Oryza *species aligned to the *O. sativa *ssp. *japonica *chromosome 1 RefSeq. The numbers in the small rectangles on the left are contig numbers of OMAP phase I physical maps. Beige bars on the right represent the *O. sativa *RefSeq (IRGSP V.4 assembly) and the red crosses on the beige bars represent the CentO position of *O. sativa *[18]. Purple lines represent BESs aligned to the *O. sativa *RefSeq. The order of the species from the left to right is; *O. nivara *[AA], *O. rufipogon *[AA], *O. glaberrima *[AA], *O. punctata *[BB]; *O. officinalis *[CC], *O. australiensis *[EE]; *O. brachyantha *[FF], *O. granulata *[GG].

Figure 1 shows a SyMAP alignment of chromosome 1 of the eight diploid *Oryza *species to the *O. sativa *RefSeq as an example. As expected, the most co-linear map alignments were observed with species that were evolutionarily less divergent (for example, AA, BB, CC). Regions of disrupted colinearity were detected mostly in the centromeric regions of the *O. sativa *genome. Although SyMAP alignments were also performed for the four polyploid species, we cannot conclude anything conclusive about their genome organization until their physical maps can be manually subdivided into each of their subgenomes.

### Sequence analysis of the OMAP BES data set: repeats, SSRs, miRNAs, and SNV

The large BES data set produced by this project offered a unique opportunity to explore sequence content and variation across the *Oryza*. We next investigated repeat and SSR content across all twelve species and identified miRNA content and SNV amongst the three AA and one BB genomes.

#### Repeats

RepeatMasker [36] in conjunction with a curated rice (*O. sativa*) repeat database was used to identify and classify previously identified repetitive elements. RECON [37] was used to identify repeats *de novo*. Results reported here relate to the number of bases covered by each element class, rather than the total number of members of each element class with the exception of small transposable elements (that is, miniature inverted repeat transposable elements (MITEs) and short interspersed elements (SINEs)), as BES reads are too short to span complete elements. The repeat content results ranged from a high of 76% in *O. australiensis *[EE] to a low of 36% in *O. coarctata *[HHKK] (Table 4). The repeats extracted by RECON overlapped with those identified by RepeatMasker by 32% in *O. coarctata *[HHKK] to 74% in *O. punctata *[BB]. This is expected as the curated repeat library used for RepeatMasker is *O. sativa *[AA] specific and, therefore, more distantly related species would be expected to share fewer repeats. Interestingly, the total repeat content of two CC genome containing allotetraploid species (*O. minuta *[BBCC] and *O. alta *[CCDD]) was less than the CC diploid genome of *O. officinalis*. It was also observed that the highest amount of unique or species-specific repetitive DNA was present in *O. australiensis *[EE] (21% corresponding to 203 Mb) and the lowest in *O. granulata *[GG] (10% corresponding to 92 Mb). These sequences were compiled into species-specific repeat databases.

**Table 4:** Repeat content analysis of 12 *Oryza *species using RepeatMasker and RECON

		Repeat content by RECON			
			
Species	Repeat content by RepeatMasker	Total	Overlap with RepeatMasker (% of total)	Uniqe (% of total)	Total repeat content
*O. sativa *(Nipponbare)	29.4%	37.2%	23.8% (64%)	13.4% (36%)	42.8%
*O. nivara*	37.0%	47.6%	29.5% (62%)	18.1% (38%)	55.1%
*O. rufipogon*	36.6%	49.7%	34.6% (70%)	15.2% (30%)	51.7%
*O. glaberrima*	28.5%	30.9%	19.8% (64%)	11.2% (36%)	39.7%
*O. punctata*	40.8%	41.4%	30.7% (74%)	10.7% (26%)	51.5%
*O. officinalis*	47.2%	56.5%	37.6% (67%)	18.9% (33%)	66.1%
*O. minuta*	44.0%	46.3%	32.2% (70%)	14.1% (30%)	58.1%
*O. alta*	43.2%	47.9%	33.2% (69%)	14.7% (31%)	57.9%
*O. australiensis*	55.0%	66.4%	45.4% (68%)	21.0% (32%)	76.0%
*O. brachyantha*	20.9%	27.5%	11.6% (42%)	15.9% (58%)	36.8%
*O. granulata*	30.1%	30.9%	20.5% (66%)	10.4% (34%)	40.5%
*O. ridleyi*	35.6%	42.7%	23.0% (54%)	19.7% (46%)	55.2%
*O. coarctata*	19.7%	24.5%	7.7% (32%)	16.7% (68%)	36.4%

The amount of repeats in each genome was directly proportional to the genome size except for *O. granulata *[GG] (Additional data file 3), suggesting that repetitive sequences play a major role in genome size expansion in the genus *Oryza*. *O. australiensis *[EE] had a high amount of repetitive DNA, consistent with the observation of a rapid burst of retrotransposons in this species [38].

**Figure 2:**
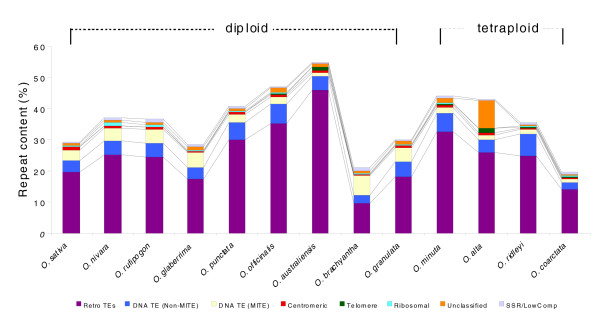
Comparison of classified repeat composition in the genomes of 12 *Oryza *species and the *O. sativa *RefSeq.

We attempted to sub-classify the repetitive elements identified by their class. Only the results from the RepeatMasker search were used for classification (Figure 2) as the *de novo *repeats identified by RECON were mostly truncated. Retrotransposons (both long terminal repeats (LTRs) and non-LTRs) comprised the major fraction of the total repeats in all species and ranged from 46% of the genome in *O. australiensis *[EE] to 9% in *O. brachyantha *[FF]. Among all types of retroelements, the Ty-3/gypsy elements were the most predominant, followed by Ty-1/copia, LINEs and SINEs, respectively. The second most abundant class was DNA (non-MITE) transposons consisting mainly of the cacta-En/Spm, mutator, mariner, ping/pong/snoopy, hAT, and Ac/Ds elements, with En/Spm being the most predominant. These non-MITE DNA transposons ranged from 7% of the genome in *O. ridleyi *[HHJJ] to 2% in *O. coarctata *[HHKK]. An exception to this trend was *O. brachyantha *[FF], which had more MITEs (7%) than DNA (non-MITE) transposons (2%). MITEs were the third most abundant elements in the genus *Oryza *with the exception of *O. brachyantha*; MITEs ranged from 0.9% of the genome in *O. australiensis *to 7% in *O. brachyantha*. The three AA genome species, as well as *O. sativa*, have approximately equal amounts of both DNA (non-MITE) transposons as well as MITEs, although with slight differences. Since MITEs represent a large class of repeats with many different types, we calculated the percentage of different MITEs from the overall total as shown in Additional data file 4. Some types of MITEs were more abundant than others and also some were specific to a particular species - for instance, the three AA genome species illustrated a similar pattern for each element. Similarly, the distribution pattern of the MITE elements to the classification of MITE types and their proportion was more comparable among *O. punctata *[BB], *O. officinalis *[CC], *O. minuta *[BBCC] and *O. alta *[CCDD] genome species rather than those of the other genomes. A similar pattern was also detected between *O. ridleyi *[HHJJ] and *O. coarctata *[HHKK] species. This result indicates that the abundance and composition of the MITE elements are closely related to genome type evolution in the genus *Oryza*.

Analysis of *O. alta *[CCDD] revealed that approximately 9% of the BESs were categorized as unclassified repeats, high relative to other 12 *Oryza *species (2% in *O. minuta *[BBCC] to 0.2% in *O. coarctata *[HHKK]), implying the existence of one or more unique classes of repeats in *O. alta *or the presence of highly diverged repeats that were recognized by our current stringency parameters. On closer inspection, we identified a copia type LTR retrotransposon in a fully sequenced BAC (OA_BBa0237I11) representing a large family of retrotransposons that accounts for the majority of these unclassified repeats. This element, considered in detail elsewhere [39], accounted for two-thirds (2,939,525 bases of 4,392,322 bases) of these unclassified repeat sequences when assayed using cross_match alone.

#### Simple sequence repeats

SSRs are extremely useful for detecting variation between species and generating molecular genetic maps. A total of 16,980 non-redundant SSRs were detected from the 1.45 million BESs analyzed, which equates to approximately 18 SSRs/Mb of *Oryza *sequence. Overall, the AA and FF genome species (*O. nivara *[AA], *O. rufipogon *[AA], *O. glaberrima *[AA] and *O. brachyantha *[FF]) showed the highest SSR content, with an average of 27 SSRs/Mb, whereas the tetraploid (*O. minuta *[BBCC], *O. alta *[CCDD], *O. ridleyi *[HHJJ] and *O. coarctata *[HHKK]) and non-AA genome diploid species (*O. punctata *[BB], *O. officinalis *[CC], *O. australiensis *[EE], and *O. granulata *[GG]) showed 17.6 and 17.2 SSRs/Mb, respectively (Table 5). Among the 16,980 non-redundant SSRs, di- and tri-nucleotide SSRs were the most abundant, representing 48.6% and 26.7% of the total dataset, respectively. The relative frequency of di-nucleotide SSRs in all the *Oryza *species was 6-7% higher than in the *O. sativa *reference genomes (Table 5; Additional data file 5). In contrast, the relative frequency of tri-nucleotide SSRs was 4-5% lower than in the *O. sativa *reference genomes and was inversely related to that of the di-nucleotide repeats in each species (Additional data file 5).

**Table 5:** Distribution of OMAP non-redundant SSRs by motif type

			Non-redundant											
			
			Di		Tri		Tetra		Penta		Hexa		Total	
								
Species	Ploidy	Total no. of SSRs containing BESs (%)	No.	%	No.	%	No.	%	No.	%	No.	%	No.	SSR density (no. of SSRs/Mb)
*O. sativa *spp *japonica*	2	-	8,636	41.81	6,259	30.31	2,439	11.81	2,628	12.72	691	3.35	20,653	55.8
*O. sativa *spp *indica*	2	-	8,317	42.94	6,059	31.28	2,285	11.80	2,142	11.06	567	2.93	19,370	47.2
*O. nivara*	2	2,129 (2.01)	739	46.33	451	28.28	189	11.85	170	10.66	46	2.88	1,595	22.5
*O. rufipogon*	2	1,599 (2.25)	696	49.40	375	26.61	169	11.99	137	9.72	32	2.27	1,409	28.2
*O. glaberrima*	2	1,289 (1.93)	518	45.00	319	27.72	124	10.77	153	13.29	37	3.21	1,151	29.5
*O. punctata*	2	1,043 (1.53)	408	44.54	268	29.26	105	11.46	100	10.92	35	3.82	916	18.7
*O. officinalis*	2	1,397 (1.35)	545	47.43	308	26.81	111	9.66	146	12.71	39	3.39	1,149	16.0
*O. australiensis*	2	1,306 (0.96)	597	54.17	230	20.87	94	8.53	128	11.62	53	4.81	1,102	13.0
*O. brachyantha*	2	1,630 (2.42)	590	48.68	287	23.68	135	11.14	166	13.70	34	2.81	1,212	26.9
*O. granulata*	2	1,174 (0.85)	603	55.99	313	29.06	64	5.94	73	6.78	24	2.23	1,077	11.6
*O. minuta*	4	1,939 (1.14)	921	54.27	386	22.75	176	10.37	168	9.90	46	2.71	1,697	17.9
*O. alta*	4	1,582 (1.23)	812	52.83	363	23.62	182	11.84	141	9.17	39	2.54	1,537	20.5
*O. ridleyi*	4	2,305 (1.13)	709	35.36	741	36.96	238	11.87	257	12.82	60	2.99	2,005	15.5
*O. coarctata*	4	2,671 (1.37)	1,108	52.02	500	23.47	257	12.07	198	9.30	67	3.15	2,130	16.5
														
OMAP total		20,064 (1.38)	8,246	48.56	4,541	26.74	1,844	10.86	1,837	10.82	512	3.02	16,980	18.2

**Figure 3:**
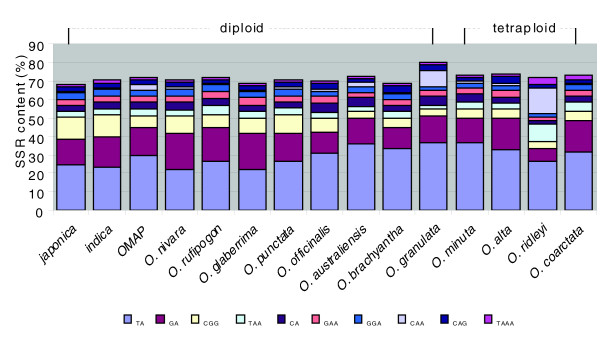
Comparison of the ten most frequent SSR motif compositions in 14 *Oryza *species (*japonica*, *O. sativa *ssp. *japonica*; *indica*, *O. sativa *ssp. *indica*; OMAP, average of 12 OMAP species).

The top ten predominant motifs from the five most frequent SSRs in each species comprised between 68% (*O. sativa *ssp. *japonica*) and 80% (*O. granulata*) of all the SSRs identified (Figure 3). The TA, GA and CGG motifs were the most abundant and accounted for a total of 51% (30%, 15%, and 6%, respectively) of all the *Oryza *SSRs. Interestingly, two SSR motifs showed biased compositions in a species-specific manner. The TAA and CAA motifs were significantly higher in *O. ridleyi *(9.6% for TAA, 13% for CAA) and *O. granulata *(9% for CAA) compared to the average (4.2% and 3%, respectively) for all *Oryza *species (Figure 3). Further investigation using the rice repeats revealed that 137 of 235 BESs (58%) containing the TAA motif in *O. ridleyi *were associated with an uncharacterized LTR retrotransposon (Additional data file 6a). We failed to detect the known interspersed rice repeats in BESs containing the CAA repeat, but a BLAST search against *O. granulata *and *O. ridleyi *BESs revealed that more than 76% of total CAA-BESs in both species hit other BESs more than 50 times. This suggests the amplification of certain repeat elements contributed to increase the TAA and CAA repeats in both species (Additional data file 6b).

The di-nucleotide SSRs showed the longest tracks of repeats, with an average of 44.5 ± 45.2 bp in the OMAP SSRs (Table 6). The TA repeat class showed the most variable repeat length among SSRs analyzed, with an average of 53.3 ± 55 bp (Additional data file 7). *O. coarctata *showed the least variable TA repeat length (31 ± 22.8 bp) and *O. rufipogon *showed the most variable length (73.8 ± 92.1 bp) in the *Oryza *species. We observed that the length of tri-nucleotide SSRs was inversely related to the G/C composition of their motifs (Additional data file 7). G/C rich motifs (CGA, CAG, CGG, TGG and GGA) revealed a shorter repeat length, with an average of 24.6 bp ± 4.5 bp in OMAP SSRs. The same trend was detected in *japonica *(24.9 ± 5.6 bp) and *indica *(24.8 ± 5.6 bp). However, A/T rich tri-nucleotide repeats (CAA, CAT, TAG, GAA and TAA) showed relatively longer tracks of repeats, with a length of 33.6 ± 18.2 bp in OMAP SSRs, 42.3 ± 30.1 bp in *japonica*, and 40.2 ± 25.9 bp in *indica*.

**Table 6:** Distribution of SSR length by the repeat types

	OMAP SSR			*japonica *SSR			*indica *SSR		
			
Repeat	No.	Average length	Standard deviation	No.	Average length	Standard deviation	No.	Average length	Standard deviation
Di	8,246	44.51	45.15	8,636	43.32	24.94	8,317	40.78	22.2
Tri	4,541	28.75	13.61	6,259	29.26	17.59	6,059	28.89	15.71
Tetra	1,844	27.69	12.69	2,439	34.25	28.81	2,285	29.9	16.51
Penta	1,837	25.11	7.73	2,628	25.24	6.39	2,142	25.52	9
Hexa	512	27.46	10.5	691	29.96	41.33	567	28.04	20.58
[C/G]A DNR*	3,151	30.29	11.08	3,617	32.31	14.76	3,722	31.55	12.45
TA DNR*	5,086	53.32	55	4,996	51.38	27.62	4,573	48.38	25.32
G/C rich TNR^†^	2,442	24.56	4.51	4,673	24.85	5.59	4,448	24.79	5.56
A/T rich TNR^†^	2,099	33.64	18.24	1,586	42.25	30.07	1,611	40.19	25.86

*Di-nucleotide repeat. ^†^Tri-nucleotide repeat.

#### Identification of miRNAs in four *Oryza *species based on OMAP alignment data

miRNAs play an important role in plant developmental and physiological processes by negatively controlling gene expression [40-43]. To detect evolutionarily conserved miRNAs between wild rice and *O. sativa *genomes, we anlayzed BES datasets from one domesticated and three wild *Oryza *genomes using an alignment-based approach.

In total, 64 paralogous miRNA precursors of *O. sativa *belonging to 22 miRNA families were identified among the four *Oryza *species (Table 7). *O. rufipogon *showed the highest number of conserved *O. sativa *miRNAs while *O. punctata *showed the lowest (22 and 7, respectively). This is consistent with our expectations since, as the evolutionary distance among species increases, the divergence within the miRNA precursor structure also increases [44]. The miRNA families of miR166 and miR171, known to play a key role in apical meristem development, were detected in all four species; the highest number of copies of each was 9 and 7, respectively. Both of these miRNAs are known to be widely shared among plant lineages. In addition, four unknown locus miRNAs (miR420, miRNA441, miR442, and miR446) appeared to be AA genome specific.

**Table 7:** miRNAs conservation between *O. sativa *and four wild rice species detected from their BES datasets

		*O. sativa *miRNA ID hits by wild rice species*				
			
*O. sativa *miRNA family	Target mRNA class	*O. nivara*	*O. rufipogon*	*O. glaberrima*	*O. punctata*	Total no. of loci
miR156	ND		d			1
miR159	MYB and TCP TFs	e	b			2
miR160	ND			d		1
miR162	DICER-LIKE 1			b		1
miR164	ND			a		1
miR166	HD-Zip TFs	f, h, k	h, k, n	h, n	c	9
miR167	Auxin response factors TFs	a, d, f	j	d	a	6
miR168	ARGONAUTE			b		1
miR169	CCAAT binding factor and HAP2-like TFs	n, p			c	3
miR171	SCARECROW-like TFs	c, e, f	c	c, d	a	7
miR319	ND				a	1
miR395	ATP sulphurylases		i, j, k			3
miR396	GRF TFs, rhodenase-like, and kinesin-like protein B			b		1
miR397	Laccases and beta-6 tubulin	a	a			2
miR399	Phosphatase TFs	e	b, e			3
miR418	ND		x		x	2
miR420	ND	x	x	x		3
miR441	ND	b	a, b, c	c		5
miR442	ND	x	x	x		3
miR446	ND	x	x	x		3
miR531	ND	x	x			2
miR535	ND	x	x	x	x	4
Total no. of loci		20	22	15	7	64

*Named miRNA loci ID [69] are shown by species (for example, a putative miR156d locus ID is noted using a 'd' in the *O. rufipogon *column). For miRNA without a locus ID (for example, miR408), an 'x' is shown. ND, not determined; TF, transcription factor.

#### Variation discovery in four wild rice species and *O. sativa*

Candidate SNVs between wild rice BESs and the *O. sativa *genome were detected with a multi-tiered alignment strategy [45] using BLASTZ [46] and cross_match followed by a base-by-base analysis of the resulting high quality alignment (see Materials and methods). Putative nucleotide variations were further filtered using sequence quality scores and quality scores of neighboring regions to exclude variations that were due to possible sequencing error.

Our initial data set contained 330,993 high quality BESs (73,716 from *O. rufipogon*, 110,589 from *O. nivara*, 73,344 from *O. glaberrima *and 73,344 from *O. punctata*). Since two assemblies of the *O. sativa *ssp. *japonica *RefSeq (International Rice Genome Sequencing Project (IRGSP) V.4 and The Institute for Genomic Research (TIGR) V.4) were available, we detected variations on both assemblies using a quality score cutoff of 23. We were able to accurately map 217,202 BESs (53,426 from *O. rufipogon*, 76,886 from *O. nivara*, 50,736 from *O. glaberrima*, and 36,154 from *O. punctata*) to the IRGSP V.4 assembly and 217,258 BESs (53,273 from *O. rufipogon*, 76,833 from *O. nivara*, 50,833 from *O. glaberrima*, and 36,319 from *O. punctata*) to the TIGR V.4 assembly. A total of 1,619,446 non-redundant SNVs (Additional data file 8a) and 319,974 insertions and deletions (INDELs; Additional data file 8b) were detected between the BESs from the four *Oryza *species (*O. rufipogon*, *O. nivara*, *O. glaberrima*, and *O. punctata*) and the *O. sativa *genome assembly from IRGSP V.4. Likewise 1,619,557 SNVs (Additional data file 8c) and 320,592 INDELs (Additional data file 8d) were detected on the TIGR V.4 assembly. The overall divergence rate was found to be 1.22%, 1.32%, 1.59% and 7.15% for *O. rufipogon*, *O. nivara*, *O. glaberrima *and *O. punctata*, respectively. The variation rate was found to be 12.2, 13.2, 15.9 and 71.5 SNVs/kb and 1.5, 1.6, 2.0 and 11.4 INDELs/kb for *O. rufipogon*, *O. nivara*, *O. glaberrima *and *O. punctata*, respectively. In comparison, the polymorphism rate reported by the Beijing Genomics Institute [47] between the *indica *and *japonica *cultivars of *O. sativa *was 1.70 SNPs/kb and 0.11 INDELs/kb [48]. Therefore, the rate of variation between *Oryza *species is roughly an order of magnitude greater than the polymorphism rate within species, as we would expect. The variation data are available through dbSNP and Gramene [49]. An example view of the variation data in Gramene is shown in Additional data file 9.

## Discussion

We describe here the generation and analysis of a comparative genomics resource for the genus *Oryza *that can be used as a research platform to address grand challenge questions in basic and applied biology. The primary resources developed were 1,452,912 BESs, 710,536 fingerprints, 12 phase I physical maps aligned to the rice RefSeq, and repeat, SSR, miRNA and SNV analyses. All sequences and variations have been deposited in GenBank and dbSNP, and all physical maps can be visualized using SyMAP [34] and CMAP at the OMAP [50] and Gramene [49] websites, respectively. The BAC libraries, BESs, SNaPshot fingerprints, and phase I physical maps have been publicly available since January 2006 and have been extensively accessed by the international community [31,38,51-56].

Estimates of genome coverage of the physical maps relative to flow cytometry data were quite close except for *O. granulate*. To make the *Oryza *physical maps more useful for future research, they must now be manually edited to merge contigs and resolve any conflicts and mis-assemblies, as was done for *O. punctata *[BB] [31]. Manual editing will be particularly challenging for the four allotetraploid species, where contigs need to be assigned to one of two subgenomes for each species. Fortunately for two tetraploids, *O. minuta *[BBCC] and *O. alta *[CCDD], we can use BES information from their diploid progenitors to assist in this process.

The BES data set represents an unprecedented sampling of genomic sequence from the *Oryza*. This resource is enhanced by the fact that the majority of BESs are binned in FPC contigs where 6-60% of BESs (depending on species) can be mapped to the rice RefSeq, and approximately 95% are paired. Here, we mined the BES data set for repeat, SSR, miRNA and SNV content. Repetitive sequences, especially transposable elements, are major components of plant genomes and contribute significantly to genome size variation. Detailed analysis of the BES resource for *O. australiensis *[EE] and *O. granulata *[GG] BESs have already demonstrated that retrotranspositional bursts of RIRE2, Wallaby and Kangaroo in the EE genome [38] and Gran3 in the GG genome [51] account for 50% and 25% of the genome sizes of these species, respectively. The RECON data described here for *O. alta *[CCDD] may likely represent yet a third retrotransposon burst accounting for genome size variation, subsequent to speciation [39].

Further analysis of the *Oryza *BES data identified 16,980 and 1,619,446 non-redundant SSRs and SNVs, respectively. The SSRs and SNVs can be immediately translated into polymorphic markers for breeding crosses between the two sequenced *O. sativa *genomes with the AA genome OMAP accessions to accelerate crop improvement. Further, the SSRs will serve as candidates to identify polymorphic genetic makers for intraspecific *Oryza *mapping populations currently under development (for example, BB × BB; CC × CC, and so on). The availability of molecular genetic maps tied to our physical maps for all 12 accessions will make these resources even stronger for functional and map-based cloning applications.

Paired-end data are also extremely important for studying structural variation, such as expansions, contractions, inversions and translocations [57,58]. Using our paired BES database, we plan to catalogue all such variation across the *Oryza *in order to address fundamental questions on the roles of such variation on the speciation process.

To our knowledge, the combined resources described herein comprise the most comprehensive within-genus comparative genomics framework available for any higher eukaryote, exceeded only by *Drosophila*, for which 12 draft genome sequences of wild flies are available [16]. We envisage that the OMAP system will be a model for other within-genus comparative frameworks, including large genome plant genera like *Gossypium *(cotton), *Solanum *(tomato), *Zea *(corn) and *Phaseolus *(soybean). Having physical map frameworks for all genome types of a particular genus aligned to a reference genome provides immediate access to any orthologous or unique region of a genome(s) for functional and evolutionary analysis. Such frameworks are also highly compatible with next generation sequencing technologies whereby whole genomes can be sequenced in 4-8 Mb chunks, thereby reducing assemblies to the size of a bacterial genome [59].

## Conclusion

The development and analysis of genus-wide research platforms is the next frontier in comparative systems biology. The genus *Oryza*, which includes the world's most important cereal, rice, is composed of 24 species (2 domesticated and 22 wild) with wide geographical distribution and contains 10 distinct genome types (6 diploid (2N = 24) and 4 polyploid (2N = 48)) and a 3.6-fold genome size variation (357-1,283 Mb).

We generated BAC-based physical maps of 12 *Oryza *species representing all the 10 genome types and aligned them to the *O. sativa *RefSeq. The 12 physical maps covered between 77% and 136% of each *Oryza *genome and revealed extensive colinearity to the *O. sativa *RefSeq. We analyzed the BES for repeats, SSRs, miRNA and SNVs. The repeat analysis not only cataloged the repeat content of each species, it suggested the possible roll they have played on genome size variation during the course of evolution. The SSR analysis identified 16,908 non-redundant SSRs from OMAP BESs, providing 18 SSRs/Mb of SSR density in the *Oryza*. We also found that the amplification of certain repeat elements contributed to an increase of the TAA and CAA motif repeats in the *O. granulata *[GG] and *O. ridleyi *[HHJJ] genomes. A total of 1,619,446 non-redundant SNVs and 319,974 insertions and deletions (INDELs) were detected between the BESs from four *Oryza *species (*O. rufipogon*, *O. nivara*, *O. glaberrima*, and *O. punctata*) and the *O. sativa *RefSeq. The SSR, SNV and INDEL markers can be immediately used for marker assistant selection as well as evolutionary studies. The miRNA analysis identified 64 paralogous miRNA precursors of *O. sativa *with more conservation in evolutionarily closer species. In addition, we observed species-wide miRNA families (miR166 and miR171) and AA genome specific miRNAs (miR420, miRNA441, miR442, and miR446).

All biological reagents are available at the AGI BAC/EST Resource Center [60] and all fingerprints and BESs are available at [49,50] and GenBank. We envision that the resources and analysis presented here will serve as a model for the establishment of similar genus-wide frameworks for plants and animals. The genus-wide physical framework also fits in well with next generation sequencing technologies where an entire genome or targeted regions across a genus can be reduced to bacterial genome size chunks that can be easily sequenced and assembled as opposed to a whole genome sequencing approach.

## Materials and methods

### BAC libraries, end sequencing, fingerprinting and FPC assembly

All BAC libraries and methods have been described previously [30,31].

### FPC map alignment to the *O. sativa *RefSeq

SyMAP [34] was used for alignment and display of FPC contigs from each of the 12 phase I physical maps to the *O. sativa *RefSeq (IRGSP V.4). Briefly, the 12 BES data sets were repeat-masked [36] and then searched for sequence similarity to the rice genome using BLAT [61]. To capture weaker sequence similarities from the more divergent species, BLAT parameters were adjusted as follows (-minIdentity = 70 -tileSize = 10 -minScore = 30 -qMask = lower -maxIntron = 10,000). BLAT results were filtered, to reduce false-positives, by retaining the top two hits for each query, as measured by match length, and subject to the additional criteria that no retained hit had a match length within 25% of a discarded hit. Approximate linear chains were computed from the retained hits using dynamic programming and then merged to form synteny blocks that were displayed by SyMAP. After the *Oryza *BESs were positioned on the *O. sativa *RefSeq, contigs containing the aligned BESs were anchored to the chromosomes and then renumbered in FPC. Contigs that aligned to less than 200 bp of *O. sativa *RefSeq were ignored in the alignment analysis (Table 3). All 12 SyMAP alignment results are available at [35].

### Synteny analysis pipeline and incorporation of data in the CMap, Genome Browser and SyntenyView displays in Gramene

We constructed a pipeline to align clones and contigs from the OMAP species to *O. sativa *by combining data from the BES alignments to *O. sativa *and the phase I physical maps. The pipeline consisted of 6 steps: 1) upload phase I physical maps and BES data, 2) align BES to the RefSeq, 3) determine the best alignments for each clone, 4) assemble the clone positions to determine the region where the contig is found to align, 5) create blocks of synteny between selected OMAP species and *O. sativa*, 6) utilize the data to create visualizations in CMap, the Genome Browser, and SyntenyView in Gramene. Details on each step can be found at [62] Screen shots of the data in Gramene are shown in Additional data file 9.

### Repeat analysis

RepeatMasker (V3.1.5), loaded with a custom *Oryza *repeat database, was used to identify repeats from the *Oryza *BES data set and the *O. sativa *RefSeq. The custom database was composed of annotated repeats from: TIGR rice repeat database [63]; Dr Susan Wessler (University of Georgia, USA); and Dr Tom Bureau (McGill University, Canada).

RECON [37] was used to identify *de novo *repeats from the *Oryza *BES data set. To increase the speed and efficiency of the program, the BLAST output was parsed to discard self hits as well as hits with an e-value greater than 1e-5. The RECON output, which identified repetitive elements and classified them into distinct families, was parsed for sequences greater than 40 bp in length that were found at least 5 times/family. Overlap between the *de novo *and the custom library was determined using RepeatMasker. Sequences left unmasked by this process and, thus, were not a part of our custom repeat database, were extracted, assembled using PHRAP [64] and annotated using BLASTN at an e-value = 1e-4 against the NCBI non-redundant nucleotide database [65] and a dataset of 2,050 full length LTR-retrotransposons identified from the whole genome sequence of Nipponbare through LTR_STRUC [66]. Finally, these sequences were compiled into repeat databases specific to each species. The procedure is outlined in Additional data file 10.

### Simple sequence repeat analysis

A total of 20,064 SSRs were identified from 1,452,912 BESs using RepeatMasker (V3.1.5) with a cutoff of longer than 20 bp and less than 5% sequence divergence. BESs containing SSRs (SSR-BES) were assembled for each species using CAP3 [67] to collect a total of 16,980 non-redundant SSRs. An SSR was considered redundant when the BAC clones came from the same FPC contig, and an assembled CAP contig contained the same SSR motif, repeat length and sequence divergence. Abundance and relative frequency of SSRs from the *Oryza *BESs were compared to SSRs identified from two rice genomic sequences: 20,653 SSRs from the *japonica *RefSeq [18] and 19,370 from the *indica *draft [68,69] sequences using identical criteria. Other interspersed repeats in SSR-BESs were detected using RepeatMasker with a custom repeat database (courtesy of Dr Ning Jiang, Michigan State University).

### Identification of *Oryza *miRNA genes

*O*.*sativa japonica *miRNA precursors from miRBase (Version 8.2) [70], including 250 nucleotides upstream and downstream, were extracted from the *O. sativa *RefSeq [18] and aligned, using BLASTN [71], against BES of four closely related wild rice species (AA and BB genomes). Alignments were filtered and multiple hits to the same region of a BES were discarded, keeping the hit with the lowest e-value. The best orthologous match typically had more conservation in the region surrounding the precursor and, therefore, had a much lower e-value. If two miRNAs aligned to different regions of the same BES, they were not discarded, since they were likely to be tandemly duplicated miRNA genes. To obtain the exact coordinates and secondary structure of the miRNA precursor within the remaining BES, a pattern matching approach was used to align the mature miRNA precursor to the BES. Once the mature miRNA was mapped to the BES, the extended sequence was then submitted to MFold to determine if it could form a stable hairpin structure [72]. MFold alignments were checked for the following criteria: had conserved orientation; contained fewer than eight mismatches; was not overlapping the loop region; and lacked some types of asymmetric bulges. A precursor was discarded if the bulge had four or more nucleotides that failed to match with any nucleotide from the opposite strand. A precursor was retained if a bulge of four or fewer nucleotides on one stem corresponded, although not necessarily matching, with one or more nucleotides on the opposite strand. If the sequence that aligned to the mature miRNA met these criteria, it was categorized as the opposing stem (miRNA*). Both stems and the intervening loop region were extracted and resubmitted as input to MFold. If this refined sequence formed a stable hairpin structure under the four conditions, it was categorized as a paralogous miRNA precursor.

### Variation discovery

BES trace files from *O. rufipogon*, *O. nivara*, *O. glaberrima *and *O. punctata *were compared to the *O. sativa *genome with BLASTZ [46] using the S1-S2 scoring method to select only the best alignment for each read. Each chromosome was then divided into 5 Mb segments and BESs aligned to each region from the previous step were re-aligned to the segment using cross_match [64] (-bandwidth 100 -alignments -discrep_lists) with variation filtering using the neighborhood quality standard (NQS) [73]. Settings for NQS were such that candidate variation in the read had a Phred quality value (Q) of at least 23, its neighboring 7 bases on either side of the candidate variation all had Phred quality values of ≥ 15 and at least 11 of the 14 neighbors matched. If a read aligned to more than one place in the genome, then only the longest alignment with the fewest SNVs was reported. To decrease the number of false positive insertions or deletions, variations were filtered out if they had a single base variation and the quality score was less than 40 or if they were multiple base variations and the quality score was less than 23. Since two assemblies of the *O. sativa *RefSeq were available (IRGSP V.4 and TIGR V.4), variations were detected using both assemblies (Additional data file 8a-d). All data have been deposited into dbSNP and can be viewed at Gramene [49].

## Abbreviations

BAC, bacterial artificial chromosome; BES, BAC end sequence; CB, consensus band; INDELs, insertions and deletions; IRGSP, International Rice Genome Sequencing Project; LTR, long terminal repeat; miRNA, microRNA; MITE, miniature inverted repeat transposable element; OMAP, *Oryza *map alignment project; RefSeq, reference sequence; SNV, single nucleotide variation; SSR, simple sequence repeat; TIGR, The Institute for Genomic Research.

## Authors' contributions

HK, LS, DW, SAJ, and RAW designed the research; HK, KC, PS, MW, DK, and JLG performed the research; WN, MB, and CS contributed analytical tools; HK, BH, YY, NG, PS, JCM, and CM analyzed the data; HK, BH, YY, NG, PS, CM, WN, LS, DW, SAJ, CS, and RAW wrote the paper.

## Additional data files

The following additional data are available with the online version of this paper. Additional data file 1 is a figure describing the SyMAP display details. Additional data file 2 is a table listing distribution of CB units from the *O. sativa *reference genome aligned contigs to each chromosome of 12 OMAP phase I physical maps. Additional data file 3 is a figure showing correlation of genome size and repeat content in the genus *Oryza*. Additional data file 4 is a figure presenting a comparison of MITE compositions in 12 OMAP genomes and the *O. sativa *genome. Additional data file 5 is a figure showing distribution of OMAP non-redundant SSR by motif types. Additional data file 6 is a table listing repeat association analysis for TAA and CAA motifs of *O. ridleyi *and *O. granulata *and BLAST analysis of CAA-BESs. Additional data file 7 is a figure comparing the SSR length and sequence compositions of OMAP SSR motifs. Additional data file 8 is a table listing SNVs and INDELs found between the BESs for *O. rufipogon*, *O. nivara*, *O. glaberrima*, *O. punctata *and the IRGSP V.4 pseudomolecules (or the TIGR V.4 pseudomolecules). Additional data file 9 is a figure describing wild rice BESs and variation data at Gramene. Additional data file 10 is a figure showing the strategy of repeat analysis and classification using the OMAP BES resources.

## Supplementary Material

Additional data file 1 SyMAP display details. Click here for file

Additional data file 2 Distribution of CB units from the *O. sativa *reference genome aligned contigs to each chromosome of 12 OMAP phase I physical maps. Click here for file

Additional data file 3 Correlation of genome size and repeat content in the genus *Oryza*. Click here for file

Additional data file 4 Comparison of MITE compositions in 12 OMAP genomes and the *O. sativa *genome. Click here for file

Additional data file 5 Distribution of OMAP non-redundant SSR by motif types. Click here for file

Additional data file 6 Repeat association analysis for TAA and CAA motifs of *O. ridleyi *and *O. granulata *and BLAST analysis of CAA-BESs. Click here for file

Additional data file 7 Comparison of the SSR length and sequence compositions of OMAP SSR motifs. Click here for file

Additional data file 8 SNVs and INDELs found between the BESs for *O. rufipogon*, *O. nivara*, *O. glaberrima*, *O. punctata *and the IRGSP V.4 pseudomolecules (or the TIGR V.4 pseudomolecules). Click here for file

Additional data file 9 Wild rice BESs and variation data at Gramene. Click here for file

Additional data file 10 The strategy of repeat analysis and classification using the OMAP BES resources. Click here for file
