# Supplementary material for: Construction, alignment and analysis of twelve framework physical maps that represent the ten genome types of the genus Oryza
Source: Genome Biol. 2008 Feb 28;9(2):R45. doi: 10.1186/gb-2008-9-2-r45 (PMC2374706; doi:10.1186/gb-2008-9-2-r45)
Supplement: Additional data file 1 — SyMAP display details. [file gb-2008-9-2-r45-S1.doc]

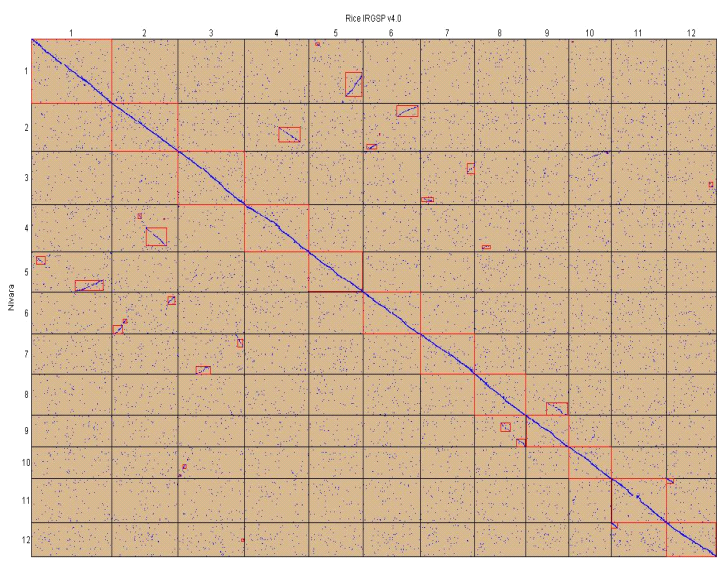

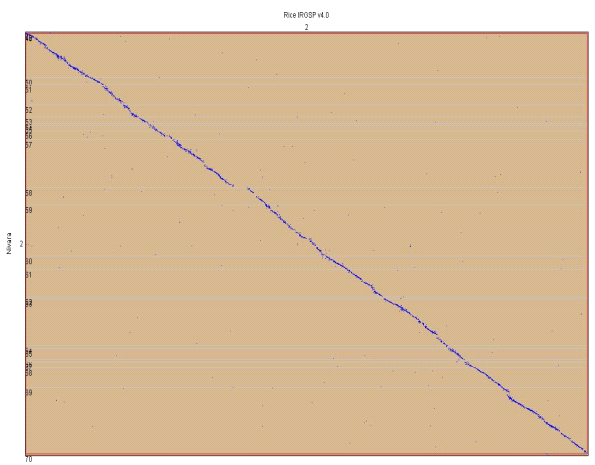

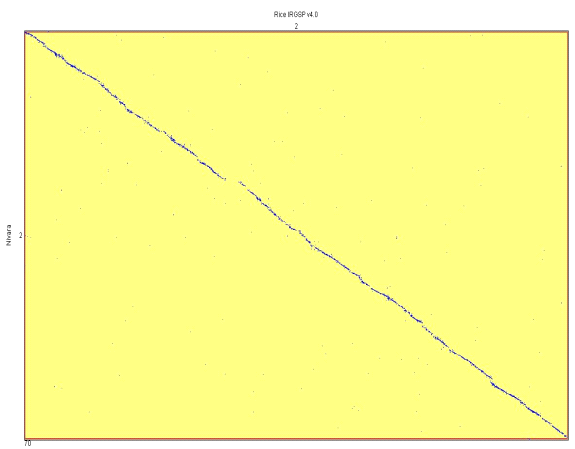

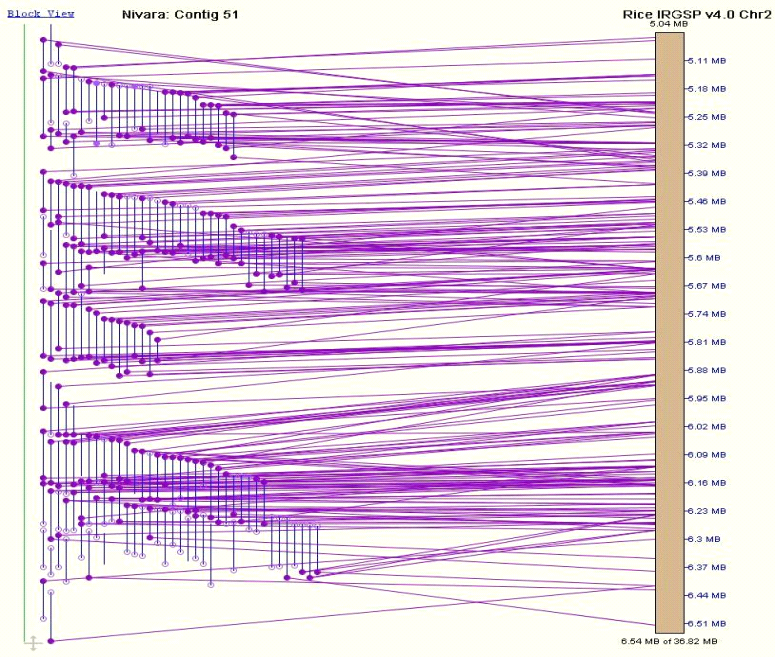

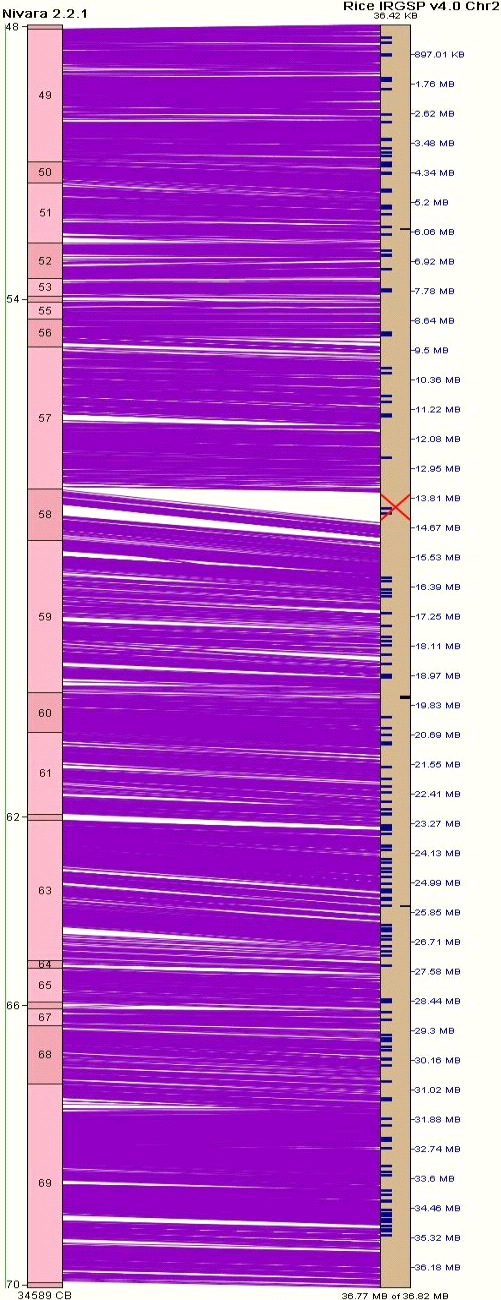

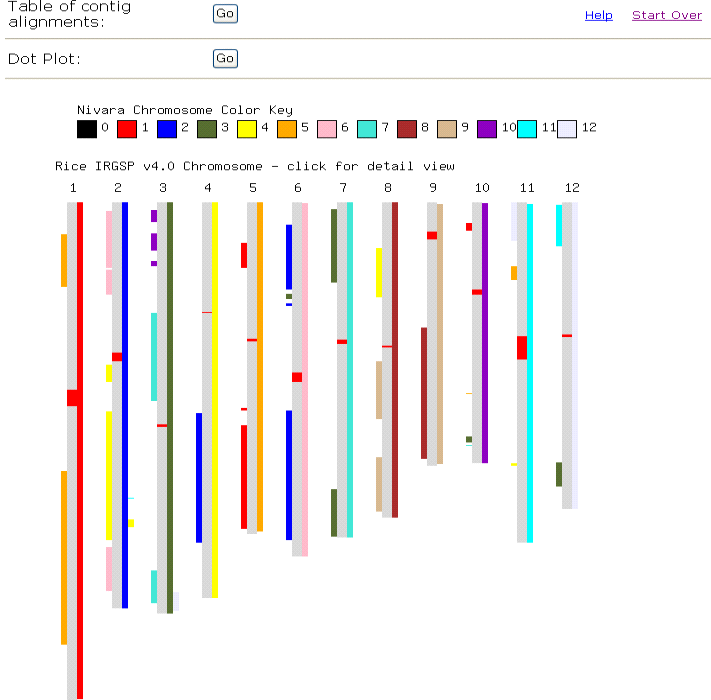


A

B

D

C


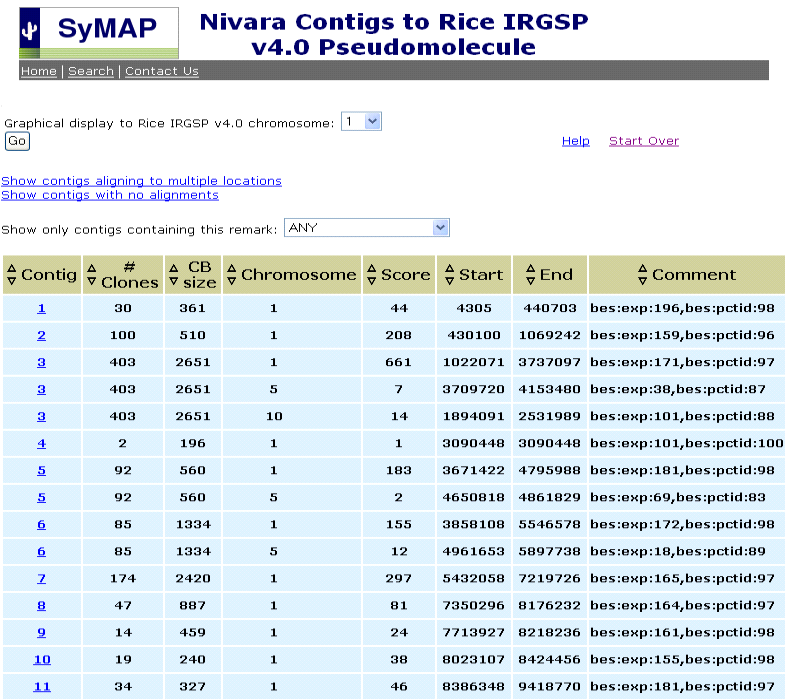


F

E

G


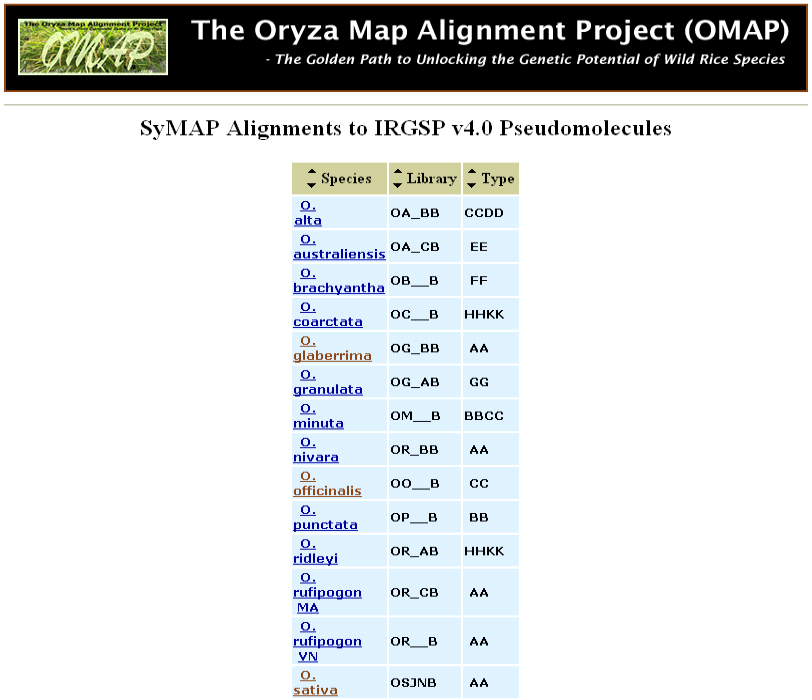


**Additional data file 1.**

 The SyMAP display details.

(A) SyMAP view of OMAP species. Clicking the species name displays SyMAP home page of each species as in (B).

(B) SyMAP generated blocks aligned to the *O. sativa* reference genome sequences of each chromosome. The blocks are color coded to indicate the FPC chromosome of origin. Clicking on an *O. sativa* chromosome will show a close-up as in (E). Clicking on the ‘dot plot’ panel displays a dot plot view as in (C). Clicking on the ‘table of contig alignment’ panel displays details of the alignment of each contig as in (D).

(C) Dot plot view of two whole genomes, the OMAP phase I physical map and the *O. sativa* reference genome sequence. The vertical axis is the OMAP phase I physical maps and the horizontal axis is the *O. sativa* reference genome sequence. Each square represents each chromosome. Red squares represent anchors shared by the two chromosomes. Clicking on a square zooms in on it. A region can be selected from within a square and then further zoomed as shown in (F)

(D) Table view of the alignment of each contig with details. Selecting a contig number shows a close-up as in (G)

(E) The close-up of a sequenced chromosome, where the numbers in the small rectangles are contig numbers of OMAP phase I physical maps. Selecting a block displays the close-up shown in (F).

(F) Close-up of a block. Purple lines are OMAP BESs aligned onto the *O. sativa* reference genome sequence. Select a contig to show the close-up seen in (G).

(G) Close-up of the contig. The displays F and G have filters for the three tracks. The Block and Contig filters allow marker and clone names to be filtered on different properties. The Hit filter allows the lines between the anchor points to be filtered on different properties. The sequence filter allows the annotation, framework markers, and gaps to be made visible/invisible. (Modified from Soderlund et al. 2006) [34].
