# Supplementary material for: Construction, alignment and analysis of twelve framework physical maps that represent the ten genome types of the genus Oryza
Source: Genome Biol. 2008 Feb 28;9(2):R45. doi: 10.1186/gb-2008-9-2-r45 (PMC2374706; doi:10.1186/gb-2008-9-2-r45)
Supplement: Additional data file 2 — Distribution of CB units from the O. sativa reference genome aligned contigs to each chromosome of 12 OMAP phase I physical maps. [file gb-2008-9-2-r45-S2.doc]

**Additional data file 2. Distribution of CB units from the *O. sativa* reference genome aligned contigs to each chromosome of 12 OMAP phase I physical maps**

The distribution of CB sizes for each chromosome among the species was fairly uniform with the standard deviation of <1.2% indicating the randomness and nonbiased coverage of our OMAP phase I physical map resources.
