# Supplementary material for: Construction, alignment and analysis of twelve framework physical maps that represent the ten genome types of the genus Oryza
Source: Genome Biol. 2008 Feb 28;9(2):R45. doi: 10.1186/gb-2008-9-2-r45 (PMC2374706; doi:10.1186/gb-2008-9-2-r45)
Supplement: Additional data file 6 — Repeat association analysis for TAA and CAA motifs of O. ridleyi and O. granulata and BLAST analysis of CAA-BESs. [file gb-2008-9-2-r45-S6.doc]

**Additional data file 6A. Repeat association analysis for TAA and CAA motif of *O. ridleyi* and *O. granulata***

**Additional data file 6B. BLAST analysis^*^ of CAA-BESs**

|  | *O. ridleyi* | *O. granulata* |
| --- | --- | --- |
| CAA-BES | 315 | 113 |
| Total hit | 105862 | 8514 |
| Unique hit | 104283 | 8453 |
| CAA-BES hit | 285 | 108 |
| Unique BES other than CAA-BESs | 1980 | 666 |
| CAA-BESs >=10 unique BES hit | 247 | 92 |
| CAA-BESs >=20 unique BES hit | 241 | 92 |
| CAA-BESs >=50 unique BES hit | 238 | 90 |

^*^CAA motif containing BESs were searched against BES database of *O. ridleyi* and *O. granulata* using BLASTN.

The blast hits with ≥ 95% of identity and ≤ 1e-50 of e-value were considered for this analysis.
