# Supplementary material for: Construction, alignment and analysis of twelve framework physical maps that represent the ten genome types of the genus Oryza
Source: Genome Biol. 2008 Feb 28;9(2):R45. doi: 10.1186/gb-2008-9-2-r45 (PMC2374706; doi:10.1186/gb-2008-9-2-r45)
Supplement: Additional data file 7 — Comparison of the SSR length and sequence compositions of OMAP SSR motifs. [file gb-2008-9-2-r45-S7.doc]

**Additional data file 7.** Comparison of the SSR length and sequence compositions of OMAP SSR motifs.
