# Supplementary material for: Construction, alignment and analysis of twelve framework physical maps that represent the ten genome types of the genus Oryza
Source: Genome Biol. 2008 Feb 28;9(2):R45. doi: 10.1186/gb-2008-9-2-r45 (PMC2374706; doi:10.1186/gb-2008-9-2-r45)
Supplement: Additional data file 8 — SNVs and INDELs found between the BESs for O. rufipogon, O. nivara, O. glaberrima, O. punctata and the IRGSP V.4 pseudomolecules (or the TIGR V.4 pseudomolecules). [file gb-2008-9-2-r45-S8.doc]

**Additional data file 8A. A list of single nucleotide variations (SNV) found between the BES for *O. rufipogon*, *O. nivara*, *O. glaberrima*, *O. punctata* and the IRGSP V.4 pseudomolecules**

Download from <ftp://ftp.genome.arizona.edu/pub/GB>

**Additional data file 8B. A list of insertions and deletions (INSDEL) found between the BES for *O. rufipogon*, *O. nivara*, *O. glaberrima*, *O. punctata* and the IRGSP V.4 pseudomolecules**

Download from <ftp://ftp.genome.arizona.edu/pub/GB>

**Additional data file 8C. A list of single nucleotide variations (SNV) found between the BES for *O. rufipogon*, *O. nivara*, *O. glaberrima*, *O. punctata* and the TIGR V.4 pseudomolecules**

Download from <ftp://ftp.genome.arizona.edu/pub/GB>

**Additional data file 8D. A list of insertions and deletions (INSDEL) found between the BES for *O. rufipogon*, *O. nivara*, *O. glaberrima*, *O. punctata* and the TIGR V.4 pseudomolecules**

Download from <ftp://ftp.genome.arizona.edu/pub/GB>
