# Supplementary material for: Construction, alignment and analysis of twelve framework physical maps that represent the ten genome types of the genus Oryza
Source: Genome Biol. 2008 Feb 28;9(2):R45. doi: 10.1186/gb-2008-9-2-r45 (PMC2374706; doi:10.1186/gb-2008-9-2-r45)
Supplement: Additional data file 9 — Wild rice BESs and variation data at Gramene. [file gb-2008-9-2-r45-S9.doc]

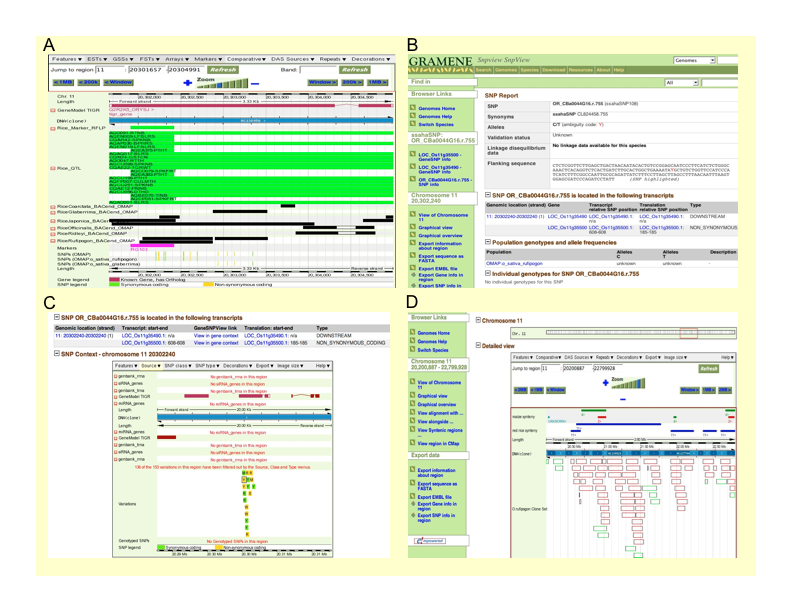


**Additional data file 9.** Wild rice BES and variation data are available via Gramene. This figure shows several web browser pages that allow researchers to view OMAP BES in the context of genes, variations, QTL regions and phenotypes of the reference species *Oryza sativa* [17]. The *Contigview* (A) shows mapped OMAP BES and sequence variations in context with mapped QTLs, genes, and other features. From here, the user can drill down to features of interest or overlap comparative data from other cereal species.

In this example, we start from the *Xa21* gene in the *Contigview* which is known to confer resistance to *Xanthamonas*. Detailed information about the gene, gene transcripts and proteins can be found by selecting *Geneview,* *Transview* or *Protview* from a drop down menu attached to the gene (not shown). For example, the transcript view,*Transview,* provides detailed information about the transcript including GO terms, similarity matches and positional information. Beneath the gene track we can see wild rice BES alignments and variations as compared to *O. sativa*. By clicking on a variation we can get detailed information about that variation in the *SNPView* (B) and or view it in the context of the gene transcript (C). The *CytoView* (D) shows details from the BAC FPC maps, and is most useful for visualizing blocks of synteny and the underlying clone evidence.   By clicking on a clone in the *CytoView* (D) we can jump to the comparative map view (CMap) where the clones are anchored to the physical maps of the wild rice species.
